# Adherence to COVID-19 Prevention Measures in the Democratic Republic of the Congo, Results of Two Consecutive Online Surveys

**DOI:** 10.3390/ijerph18052525

**Published:** 2021-03-04

**Authors:** John D. Ditekemena, Hypolite M. Mavoko, Michael Obimpeh, Stijn Van Hees, Joseph Nelson Siewe Fodjo, Dalau M. Nkamba, Antoinette Tshefu, Wim Van Damme, Jean Jacques Muyembe, Robert Colebunders

**Affiliations:** 1 Kinshasa School of Public Health, Faculty of Medicine, University of Kinshasa, Kinshasa 834, Democratic Republic of the Congo; dalau.nkamba@unikin.ac.cd (D.M.N.); antoinette.tshefu@unikin.ac.cd (A.T.); 2 Department of Tropical Medicine, Faculty of Medicine, University of Kinshasa, Kinshasa 834, Democratic Republic of the Congo; hypolite.muhindo@unikin.ac.cd; 3 Global Health Institute, University of Antwerp, 2610 Antwerp, Belgium; Michael.Obimpeh@student.uantwerpen.be (M.O.); Stijn.VanHees@uantwerpen.be (S.V.H.); JosephNelson.SieweFodjo@uantwerpen.be (J.N.S.F.); robert.colebunders@uantwerpen.be (R.C.); 4 Pôle d’Épidémiologie et Biostatistique, Université Catholique de Louvain (UCLouvain), Institut de Recherche Expérimentale et Clinique (IREC), 1348 Brussels, Belgium; 5 Department of Public Health, Institute of Tropical Medicine, 2600 Antwerp, Belgium; wvdamme@itg.be; 6 Institut National de Recherche Biomédicale and Faculty of Medicine, University of Kinshasa, Kinshasa 834, Democratic Republic of the Congo; jjmuyembet@gmail.com

**Keywords:** COVID 19, adherence, prevention measures, infectious diseases

## Abstract

Adherence to preventive measures is essential to reduce the risk of COVID-19 transmission. Two online surveys were conducted in the Democratic Republic of the Congo (DRC) from 23 April to 8 June 2020, and from August 24th to September 8th, respectively. A total of 3268 (round 1) and 4160 (round 2) participants were included. In both surveys, there was a moderate level of adherence to regular handwashing (85% and 77%, respectively), wearing of facemasks (41.4% and 69%, respectively), and respecting physical distancing (58% and 43.4%, respectively). The second survey found that, working in private (OR = 2.31, CI: 1.66–3.22; *p* < 0.001) and public organizations (OR = 1.61, CI: 1.04–2.49; *p* = 0.032) and being a healthcare worker (OR = 2.19, CI: 1.57–3.05; *p* < 0.001) significantly increased the odds for better adherence. However, a unit increase in age (OR = 0.99, CI: 0.98–0.99; *p* < 0.026), having attained lower education levels (OR = 0.60, CI: 0.46–0.78; *p* < 0.001), living in a room (OR = 0.36, CI: 0.15–0.89; *p* = 0.027), living in a studio (OR = 0.26, CI: 0.11–0.61; *p* = 0.002) and apartment (OR = 0.29, CI: 0.10–0.82; *p* = 0.019) significantly decreased the odds for better adherence. We recommend a multi-sectorial approach to monitor and respond to the pandemic threat. While physical distancing may be difficult in Africa, it should be possible to increase the use of facemasks.

## 1. Introduction

The SARS-Cov 2 virus was first reported in the city of Wuhan, Hubei Province, China, in December 2019. It reached several countries on all continents and was declared a pandemic in March 2020 [1,2,3]. Non-pharmaceutical interventions such as physical distancing, facial mask use, hand sanitizer use, and regular handwashing have been recommended and implemented in nearly all countries [4,5,6]. In addition, the majority of countries have established containment and lockdown measures, with bans on all events with crowds and international travel restrictions. These measures decreased the number of new infections, hospitalization needs, and COVID-19 related mortality and led leaders around the world in July and August to relax measures, including containment, international travel, and bans on social events [7].

Around October 2020, a second wave of the pandemic surfaced in several countries already heavily affected during the first wave. As of 13 January 2021, the number of COVID-19 new infections was 94.5 million worldwide, with 2.0 million as the cumulative number of deaths in the world [2,8,9]. A lot of effort has been put into the development of an appropriate response to the pandemic. Several vaccine candidates are in advanced stages. In November 2020, some vaccine candidates were announced to be effective, and by December 2020, a vaccination campaign had been launched in several countries around the world [10,11,12].

Thus far, the DRC has reported only a limited number of COVID-19 cases. Several fears arose at the onset of the COVID-19 pandemic regarding the DRC as a potential pool of worsening epidemics [13,14]. Nonetheless, despite limitations for COVID-19 testing in the country, the number of complicated and severe COVID-19 cases has not been high or alarming, without a clear explanation thus far [2]. On 13 January 2021, the DRC reported 21,059 cumulative cases of COVID-19 infections and only 633 of cumulative deaths for an estimated population of more than 80 million people. Investigating how well the general population in the DRC observed the COVID-19 preventive measures might provide some reasons behind this unexpectedly low reported COVID-19 burden. A first online survey to assess adherence to COVID-19 preventive measures recommended by the World Health Organization (WHO) and the local government was conducted in the DRC from 23 April and 8 June 2020 during the lockdown period [15]. A second survey was conducted from 24 August to 8 September 2020 just after the lockdown. In the meantime, social mobilization efforts had been undertaken by WHO local and regional offices, international organizations, national public health agencies, and the DRC government authorities to promote uptake and adoption of the prescribed COVID-19 prevention measures. The aim of the second survey was to describe the evolution of the level of adherence as well as factors associated with good and poor adherence.

## 2. Materials and Methods

### 2.1. Study Setting and Design

The DRC is the largest country in the Central African region, with an estimated population of 89 million, a total fertility rate of 6 children born per woman, and a life expectancy of 61.6 years. The population of the DRC is relatively young: 62.7% are between 0–24 years, 30.9% between 25–54 years, and 6.3% ≥55 years of age. The country is composed of 26 provinces and shared borders with 9 countries. In terms of the operational health system structure, the DRC has 516 health zones [15].

The country has a recent history of armed civil unrest and is currently confronted with residual armed conflicts in the eastern part of the country. This country experienced recent outbreaks of vaccine-preventable diseases, including measles, polio, cholera, and yellow fever. The country is ranked second in Africa in terms of tuberculosis burden, is endemic for malaria, and has a 1.2% HIV prevalence. Damage to and depletion of the DRC’s rainforests exacerbated by war-related displacement of the population to forests, poaching, illegal lumber trade, and artisanal mining continue to precipitate episodic contact of people with the animal reservoirs of other viruses, including Ebola and Monkeypox [14,15,16]. The eastern part of the DRC has experienced a long-running Ebola virus epidemic since August 2018. Insecurity due to armed rebel militias made the control of Ebola difficult. Such a context should be well monitored for the region and global health interest and security with regards to COVID-19 pandemic consequences. The DRC government decided to extend the mandate of the National Ebola task committee to COVID-19 management to take advantage of long Ebola management experience for COVID-19 management. Already early, on 18 March 2020, despite very few COVID-19 cases, the DRC decided to close its borders for incoming travelers and closed churches, markets, bars, restaurants, and dance clubs, where super-spreading events could take place. As of July 2020, the COVID-19 epidemic in the DRC was still mainly concentrated in Kinshasa, especially in La Gombe commune and a few bordering areas. With an R0 of 3, the virus spread in the provinces, but with few confirmed cases outside Kinshasa. A 2nd wave of COVID 19 appeared around November and December 2020. During this 2nd wave, the DRC government imposed a curfew from 9 pm until 5 am, with mandatory preventive measures such as the use of masks and physical distancing.

We conducted 2 cross-sectional online surveys, the 1st during the lockdown period and the 2nd just after the lockdown. Both surveys were part of a series of surveys organized by an International Consortium (International Citizen Project COVID-19 (ICPCovid); www.icpcovid.com, accessed on 20 January 2021) in Low and Middle Income Countries (LMICs) to monitor the degree to which people aged ≥18 years adhere to COVID-19 preventive measures.

### 2.2. Study Instrument and Participants’ Recruitment

A web-based online questionnaire (see Supplementary Material) using the ICPcovid website https://www.icpcovid.com/ (accessed on 20 January 2021) was used. The questionnaire proposed by the ICPCovid consortium was translated from English to French, adapted, pre-tested, and used during the 1st round of the online survey in the DRC. [15] The survey instrument included questions on demographic characteristics, including age, sex, educational level, and occupation. In addition, the questions about the presence or absence of flu-like symptoms during the preceding 14 days, the specific symptom(s) they experienced, whether they had been tested for COVID-19, were also asked. Our data were reported according to Reporting Results of Internet E-Surveys (CHERRIES) guidelines.

The link for the questionnaire was disseminated online to as many people as possible in the 17 provinces of the DRC via social media platforms such as Facebook and by using WhatsApp and emails. Upon clicking on the link, the potential participant was informed about the study objectives, data confidentiality, and consent form. We used 1 to 4 study assistants in each province to increase participation to assist potential study candidates who had no access to the internet or had difficulties in filling out the form [15]. Study assistants were asked to motivate potential participants in their network to participate in the survey. In addition, they were asked to interview the first 60 people they met per day in a targeted street. Transportation and mobile internet fees were reimbursed to the study assistants. The study participants did not receive any financial support or incentive. Information from the participants was directly recorded by the participants. In some instances, and where needed, the study assistants shared their internet access to enable participants to access the online questionnaire. Convenience and snowball sampling methods were used. Either surveyors themselves contacted potentials participants in different districts, or the participants were requested to share the link of the questionnaire with their contacts [15].

### 2.3. Assessment of Adherence

General adherence to the COVID-19 preventive measures was assessed using items configured as yes/no questions in the questionnaire. A composite adherence score was made by summing responses to the mentioned 10 questions (Table 1) and recoded as Low adherence (0–5 points), Moderate adherence (6–7 points), and high adherence (8–10 point). Subsequently, we categorized moderate and high adherence (adherence ≥ 6) into one category and coded it adherence (1), while inadequate practices (adherence < 6) were considered as non-adherence (0) to COVID-19 preventive measure.

### 2.4. Data Processing and Analysis

Three respondents who reported to be less than 10 years old were excluded from the data set. Descriptive statistics were presented using means and standard deviation (SD) for continuous variables and percentages for categorical variables. Continuous variables were compared across groups using Kruskal–Wallis test as appropriate and categorical variables were compared across groups using a chi-squared test as appropriate.

The dependent variable used was adherence score and was dichotomized into adherence versus non-adherence to the preventive measures.

In the bivariate analysis, we selected factors that could be associated with the level of adherence. All variables with a likelihood ratio *p*-value < 0.25 in bivariate regression were included in the multivariable analysis. The selected variables from the bivariate analysis were subjected to a backward stepwise selection process, and a final model with the least Akaike information criterion (AIC) was selected.

Multivariable analysis was conducted to investigate factors associated with adherence to national preventive measures against COVID-19. Logistic regression was conducted with Generalized Estimation Equations (GEE) to control for correlation among study participants in the same province. We adopted the exchangeability assumption for the correlation structure even though GEEs were robust to misspecifications of the correlation structure [17] within each province, hence the cluster effect was controlled for each province. We also estimated the variance inflation factors to check for multi-collinearity, and this was negligible since these values were less than 10 as a rule of thumb. The level of significance used was 5%, and all tests were 2 sided. The relationship between the dependent and independent variables was determined by adjusted odds ratios (AOR), with 95% confidence intervals (95% CI) and *p*-value < 0.05 to determine the statistical significance level of these factors.

The level of worry and fear concerning a participant’s health in the midst of COVID-19 pandemic (scored on a 5-point Likert scale) was also analyzed. Group-specific means and standard deviations were presented for significant characteristics using the Kruskal-Wallis test.

We determined the proportions of participants across the 2 surveys that reported flu-like symptoms that met the WHO COVID-19 diagnostic criteria as well the criteria of the newly proposed case definition, which included anosmia or ageusia [18,19]. We also reported the total number of laboratory-confirmed cases of COVID-19 and compared them across the 2 surveys using the Pearson chi-squared test. Statistical analysis was performed using R software version 4.0.3.

### 2.5. Ethical Considerations

The study protocol was submitted and approved by the DRC National Ethics Committee. To ensure confidentiality, data were collected online anonymously and were only available to study investigators using passwords. The study investigators were certified on ethical training as well as good clinical practices, and they were bound by professional secrecy with regard to all the information collected during this study. All participants provided an e-consent before submitting their responses.

## 3. Results

### 3.1. Participant’s Characteristics of the First and Second Survey

There was a total of 3268 and 4160 participants in the first and second surveys, respectively. Ten provinces were excluded from the second survey analysis because they had less than 350 respondents [15], this excluded 29 respondents. Thus, 4131 participants were included in the analysis from 7 provinces: Haut Katanga, Kasaï Central, Kasaï Oriental, Kinshasa, Congo Central, Kwilu, and North Kivu. In both surveys, the highest percentage of participants were in the 18–30 and 39 –49 years’ age groups (Table 2). Female participants represented 66% and 68% of the respondents in Survey 1 and Survey 2, respectively. Most participants in both surveys had a secondary level educational level (53% and 65% respectively) and were legally married (60% and 55%, respectively). Religious groups were equally represented in both surveys (Table 2). Only a small percentage of the participants were healthcare workers in the first (10%) and second survey (8%).

### 3.2. Level of Adherence

In both surveys, there was a high level of adherence to regular handwashing (85% and 77%, respectively) and the wearing of facemasks (41.4% and 69%, respectively). However, there was a slight reduction in the percentage of participants who respected the minimum 1.5 m physical distance rule (from 58% in the first survey to 43.4% in the second survey) and of participants who stayed at home when they experienced flu-like symptoms (from 61% in the first survey to 47% in the second survey) (Table 3).

### 3.3. Level of Worry and Fear Concerning Participant’s Health

In both surveys, respondents who reported practicing the Adventist religion and those who lived in huts were considerably more worried about their own health (Table 4).

### 3.4. Prevalence of Suspected COVID-19 Infection

Applying the WHO’s clinical definition and a recently proposed case definition for COVID-19 screening (without taking into account a history of contacts) [18,19], the prevalence of suspected COVID-19 cases ranged from 6.5% to 7.4% in the first survey, and 9.1% to 10.0% in the second survey. The proportion of persons who had ever had a test for COVID-19 increased from 1.1% in the first survey to 10.7% in the second survey. However, among respondents who had been tested within two weeks prior to submitting their responses to the surveys, 8.6% and 8.0% tested positive in the first and second survey, respectively. (Table 5).

### 3.5. Factors Associated with Adherence to COVID-19 Preventive Measures

A logistic regression model with GEE estimation procedure investigating factors associated with adherence to COVID-19 preventive measures found that, working in private (OR = 2.31, CI: 1.66–3.22; *p* < 0.001) and public organizations (OR = 1.61, CI: 1.04–2.49; *p* = 0.032) and being a healthcare worker (OR = 2.19, CI: 1.57–3.05; *p* < 0.001) significantly increased the odds for better adherence. However, a unit increase in age (OR = 0.99, CI: 0.98–0.99; *p* < 0.026), having attained lower education levels (OR = 0.60, CI: 0.46–0.78; *p* < 0.001), living in a room (OR = 0.36, CI: 0.15–0.89; *p* = 0.027), living in a studio (OR = 0.26, CI: 0.11–0.61; *p* = 0.002), and apartment (OR = 0.29, CI: 0.10–0.82; *p* = 0.019) significantly decreased the odds for better adherence (Table 6).

## 4. Discussion

Overall adherence to COVID-19 preventive measures (10-point score) was similar between the two surveys, but adherence was not good for certain measures such as facemask use and physical distancing. Adherence to the physical distancing rule decreased between both surveys, while the use of masks increased. Less adherence with the physical distancing rule was to be expected after the end of the lockdown measures, while increased mask use may have been the result of sensitization activities conducted by health services and the COVID-19 National Task Force. In addition, the use of the police by the Government of the DRC may have encouraged people to wear masks to avoid penalties. It is possible that for fear of reprisals by the police, some people have become accustomed to wearing a mask. In addition, through local manufacturing, facemasks have become more readily available [20,21]. Our survey shows the difficulty of respecting the physical distancing rule in an African setting. Therefore, increasing the use of facemasks is very important. The 69% of facemask use in the DRC (and this is most likely an overestimate of the real percentage) is still too low and should be increased to curb COVID-19 transmission effectively. Indeed, in other African countries such as Mozambique, a similar survey found that more than 90% of respondents reported wearing facemasks [22].

The use of regular handwashing decreased between the two surveys. One of the reasons for this may be the decrease in the number of handwashing stands in front of shops and supermarkets (HM Mavoko, personal observation). Likewise, the use of hydro-alcoholic gel also decreased from 52% to 41%. The proportion of respondents that reported they would stay at home if they had flu-like symptoms decreased over time from 61% in the first survey (during the confinement period with risk to be quarantined) to 47% in the second survey (confinement lifted). Being a Seventh-day Adventist and living in a hut was associated with a high level of fear for their health.

Our survey suggests that since the first survey in April–June 2020, after de-confinement with resumption of activities such as local and international travels, COVID-19 transmission increased. Indeed, the prevalence of suspected COVID-19 infection using the WHO definition increased from 6.5% in the first survey to 9.1% in the second survey. Fortunately, the COVID-19 testing capacity in the DRC has also ramped up with time as the number of respondents who reported to have been tested for COVID-19 during the two weeks preceding the survey increased from 35 during the first survey to 441 during the second survey (Table 5). Although more people were tested during the second survey period, the proportion of positive tests among respondents did not differ much from the first survey (8.6% vs. 8.0%).

In the second survey, we identified several factors that were associated with adherence to preventive measures. A higher education level was associated with a higher adherence score [15,23]. Compared to students/unemployed respondents, working in a private or public company was associated with a high adherence score. In these settings, adherence to preventives measures is required. In addition, the availability of sensitization posters educating workers may have played a role [24].

Living in a single room or a small studio/apartment was associated with a low adherence score. This may be related to the lower socioeconomic conditions of the respondents. It is possible that with limited accommodation space, residents would find it difficult to respect social distancing between themselves, and if conditions are precarious, even the availability of masks and other products like hydro-alcoholic hand sanitizers would be compromised as well. We should also consider the possibility that some respondents who reported living in single rooms may not have any housemates. However, such habitats still depict relatively low living standards even for those who reside there alone. Several studies have shown a correlation between precarious socioeconomic conditions and decreased adherence to health measures [25,26,27].

Healthcare workers were more adherent to the preventive measures. This is a very positive finding as healthcare workers are in charge of the sensitization of communities about COVID-19 preventive measures and are expected to set an example [15,28,29].

It is important to note the limitations of our study. Indeed, the participants in our survey cannot be considered representative of the Congolese population. Persons with a higher educational level and living in cities were overrepresented. Moreover, self-reported responses may not reflect the real-life behavior of participants. Our results show that adherence of the respondents to the COVID-19 preventive measures was sub-optimal, but the real adherence of the Congolese population to these measures is certainly worse. Indeed, very little mask use is observed in busy parts of big cities, and many people are not respecting physical distancing at marketplaces and in busses.

## 5. Conclusions

Overall there was moderate adherence to COVID-19 preventive measures in the DRC without major changes over time. Facemask use remained low and physical distancing was often not respected. While respecting physical distancing may be difficult in an African setting, it should be possible to increase the use of facemasks. Factors such as housing conditions, employment, and educational level were found to affect adherence to the preventive measures. Therefore, we recommend implementing a multi-sectorial approach to monitor and respond efficiently to the pandemic threat with regards to the second or third pandemic wave. In the context of the DRC, some influential stakeholders, including education sector leaders, religious leaders, traditional media leaders, and traditional leaders, should be involved in the COVID-19 response to increasing the efficiency of a prevention campaign.

## Figures and Tables

**Table 1:** Composition of General Adherence score (0–10).

Variable	Scoring		Interpretation
Observing 1.5–2 m physical distance rule	Yes	1	1 point for yes, 0 point for no
	No	0	
Wearing a face mask when going out	Yes	1	1 point for yes, 0 point for no
	No	0	
Washing hands regularly	Yes	1	1 point for yes, 0 point for no
	No	0	
When I cough/sneeze, I cover my mouth and nose	Yes	1	1 point for yes, 0 point for no
	No	0	
Avoid touching my face (eyes, nose, mouth)	Yes	1	1 point for yes, 0 point for no
	No	0	
Did you travel within the last 7 days	Yes	0	1 point for no, 0 point for yes
	No	1	
Disinfecting phone when getting home;	Yes	1	1 point for yes, 0 point for no
	No	0	
Using alcohol-based hand sanitizer during the day	Yes	1	1 point for yes, 0 point for no
	No	0	
Hands washing/disinfecting right after coughing or sneezing;	Yes	1	1 point for yes, 0 point for no
	No	0	
Staying at home when having flu-like symptoms	Yes	1	1 point for yes, 0 point for no
	No	0	
Total Adherence Score (Maximum): 10			

**Table 2:** Participants’ characteristics in the first and second survey.

Characteristics	Survey 1 (*n* = 3268)	Survey 2 (*n* = 4131)	*p*-Value
**Age groups**			
18–29 years, *n* (%)	1300 (40%)	1521 (36.8%)	0.004
30–39 years, *n* (%)	834 (25%)	1321 (32.0%)	
40–49 years, *n* (%)	620 (19%)	786 (19%)	
50 + years, *n* (%)	514 (16%)	503 (12.2%)	
**Sex**			
Female, *n* (%)	2173 (66%)	2827 (68.4%)	0.106
Male, *n* (%)	1095 (34%)	1304 (31.6%)	
**Nationality**			
Congolese, *n* (%)	3221 (99%)	4069 (98.5%)	0.929
Foreigner, *n* (%)	47 (1%)	62 (1.5%)	
**Religion**			
Muslim, *n* (%)	70 (2%)	135 (3.3%)	<0.001
Catholic, *n* (%)	1274 (39%)	1553 (37.6%)	
Protestant, *n* (%)	590 (18.1%)	643 (15.6%)	
Pentecostal, *n* (%)	499 (15.3%)	484 (11.7%)	
Seventh Day Adventist *n* (%)	84 (2.6%)	172 (4.0%)	
Jehovah witness, *n* (%)	145 (4.4%)	176 (4.3%)	
Other, *n* (%)	568 (17.4%)	848 (20.5%)	
None, *n* (%)	38(1.2%)	120 (3%)	
**Education**			
University Postgraduate Degree, (M.Sc. and PhD), *n* (%)	182 (5.4%)	137 (3%)	<0.001
Tertiary (Certificate, diploma and degree), *n* (%)	1206 (37%)	1063 (26%)	
Secondary, *n* (%)	1727 (53%)	2670 (65%)	
Primary, *n* (%)	153 (4.6%)	261 (6%)	
**Marital status**			
Cohabitation, *n* (%)	290 (9%)	534 (13%)	<0.001
Divorced, *n* (%)	44 (1.3%)	87 (2%)	
Legally married, *n* (%)	1969 (60%)	2266 (55%)	
Single, *n* (%)	846 (26%)	1127 (27%)	
Widow/widower, *n* (%)	119 (3.6%)	117 (3%)	
**Occupation**			
Jobless/student, *n* (%)	1776 (54.3%)	2255 (54.6%)	0.267
Work for a person, institution, or company, *n* (%)	1057 (32.3%)	1375 (33.3%)	
Work for the government, *n* (%)	435 (13.3%)	501 (12.1%)	
**Being a healthcare worker**			
No, *n* (%)	2859 (87%)	3683 (89%)	0.014
Yes, *n* (%)	320 (10%)	324 (8%)	
Student in health sector, *n* (%)	89 (3%)	124 (3%)	
**Housing/Living Conditions**			
Homeless, *n* (%)	3 (0.1%)	21 (0.5%)	<0.001
Hut, *n* (%)	180 (5.5%)	71 (1.7%)	
Room, *n* (%)	150 (4.5%)	233 (5.6%)	
Villa, *n* (%)	548 (17%)	616 (15%)	
Studio, *n* (%)	1194 (36.5%)	1407 (34%)	
Apartment, *n* (%)	1193 (36.4%)	1783 (43%)	

**Table 3:** Adherence to preventive measures during the first and second surveys.

Adherence to Preventive Measures	Survey 1 (*n* = 3268)	Survey 2 (*n* = 4131)	*p*-Value
**Respect physical distancing**			
No, *n* (%)	1364 (42%)	2340 (56.6%)	<0.001
Yes, *n* (%)	1904 (58%)	1791 (43.4%)	
**Regular handwashing**			
No, *n* (%)	501 (15%)	967 (23.4%)	<0.001
Yes, *n* (%)	2767 (85%)	3164 (76.6%)	
**Wear facemask outside**			
No, *n* (%)	1916 (58.6%)	1274 (31%)	<0.001
Yes, *n* (%)	1352 (41.4%)	2857 (69%)	
**When I cough or sneeze I always disinfect my hands immediately**			
No, *n* (%)	2081 (64%)	2620 (63.4%)	0.734
Yes, *n* (%)	1187 (36%)	1511 (36.6%)	
**Did you travel within the last 7 days**			
No, *n* (%)	3179 (97%)	3848 (93%)	<0.001
Yes, *n* (%)	89 (3%)	283 (7%)	
**I stay at home if I feel flu-like symptoms**			
No, *n* (%)	1277 (38%)	2177 (53%)	0.005
Yes, *n* (%)	1991 (61%)	1954 (47%)	
**Regular use of alcohol-based gel**			
No, *n* (%)	1573 (48%)	2449 (59%)	0.001
Yes, *n* (%)	1695 (52%)	1682 (41%)	
**Disinfecting phone when getting home**;			
No, *n* (%)	2675 (82%)	3507 (85%)	0.005
Yes, *n* (%)	593 (18%)	624 (15%)	
**When I cough/sneeze, I cover my mouth and nose**			
No, *n* (%)	1134 (35%)	1793 (43%)	0.001
Yes, *n* (%)	2134 (65%)	2338 (57%)	
**Avoid touching my face (eyes, nose, mouth)**			
No, *n* (%)	1388 (42.5%)	2301 (56%)	<0.001
Yes, *n* (%)	1880 (57.5%)	1830 (44%)	
**General Level of adherence to preventive measures**			
Poor adherence *n* (%)	1972 (60%)	2858 (69%)	0.005
Moderate adherence, *n* (%)	464 (14%)	685 (17%)	
High adherence, *n* (%)	832 (26%)	588 (14%)	

**Table 4:** Level of worry and fear concerning participant’s health in the midst of the COVID-19 pandemic (scored on a 5-point Likert scale).

Variables	Response	Participants with Good Adherence, *n* (%)	Survey 1	*p*-Value *	Participants with Good Adherence, *n* (%)	Survey 2	*p*-Value *
			Mean Likert Score (SD)			Mean Likert Score (SD)	
Marital Status	Cohabitation	78 (27%)	2.60 (1.52)	<0.001	137(25.7%)	2.47 (1.45)	0.045
	Divorced	23 (52.3%)	2.80 (1.66)		42 (48.3%)	2.62 (1.40)	
	Legally married	673 (34.2%)	2.50 (1.62)		605 (26.7%)	2.47 (1.50)	
	Single	487 (57.6%)	2.49 (1.57)		463 (41.1%)	2.35 (1.42)	
	Widow/widower	35 (29.4%)	2.08 (1.44)		26 (22.2%)	2.74 (1.45)	
Religion	Muslim	32 (45.7%)	2.30 (1.50)	<0.001	51 (37.8%)	2.42 (1.37)	<0.001
	Catholic	494 (38.8%)	2.36 (1.53)		361 (23.2%)	2.36 (1.44)	
	Protestant	364 (61.7%)	2.60 (1.60)		230(35.8%)	2.91 (1.56)	
	Pentecostal	136 (27.3%)	2.80 (1.80)		124 (25.6%)	2.37 (1.44)	
	Seventh Day Adventist	30 (35.7%)	3.06 (1.76)		68 (39.5%)	3.02 (1.40)	
	Jehovah witness	58 (40%)	2.33 (1.54)		87 (49.4%)	2.45 (1.34)	
	Other	164 (29%)	1.91 (1.25)		308 (36.3%)	2.18 (1.43)	
	None	18 (47.4%)	2.24 (1.46)		44 (36.7%)	2.56 (1.44)	
Occupation	Company	231 (41.1%)	2.33 (1.53)	<0.001	245 (53%)	2.58 (1.50)	<0.001
	Government	277 (63.7%)	2.55 (1.60)		277 (55.3%)	2.86 (1.43)	
	Self-employed	311 (62.8%)	2.80 (1.56)		177 (19.4%)	2.39 (1.39)	
	Student	187 (42.5%)	1.95 (1.35)		257 (36.1%)	2.30 (1.39)	
	Jobless	290 (21.7%)	2.37 (1.61)		317 (20.5%)	2.38 (1.53)	
Housing/Living Conditions	Homeless	1 (33%)	1.67 (1.15)	<0.001	15 (71.4%)	2.52 (1.30)	<0.001
	Hut	111 (61.7%)	3.83 (1.71)		31 (43.7%)	3.20 (1.28)	
	Room	63 (42%)	2.07 (1.46)		72 (31%)	2.56 (1.39)	
	Villa	388 (61.7%)	2.47 (1.60)		264 (43%)	2.21 (1.38)	
	Studio	233 (19.5%)	1.94 (1.26)		314 (22.3%)	2.08 (1.30)	
	Apartment	550 (46.1%)	2.66 (1.64)		577 (32.4%)	2.78 (1.56)	

* Kruskal–Wallis test.

**Table 5:** Proportion of suspected and confirmed COVID-19 infected respondents according to the clinical definitions.

Description	Survey 1 (*n* = 3268)	Survey 2 (*n* = 4131)	*p*-Value *
Meeting WHO case definition ^1^, *n* (%)	211 (6.50%)	376 (9.10%)	<0.001
Meeting new case definition ^2^, *n* (%)	241 (7.40%)	408 (10.00%)	<0.001
Anosmia, *n* (%)	24 (0.73%)	61 (1.48%)	0.004
Ageusia, *n* (%)	69 (2.11%)	90 (2.17%)	0.910
Tested for COVID-19, *n* (%)	35 (1.07%)	441 (10.66%)	<0.001
Positive	3/35 (8.6%)	35/441 (8.00%)	0.925
Negative	32/35 (91.4%)	372/441 (84.35%)	

^1^ Individuals with fever AND at least one respiratory symptom (dry cough, productive cough, shortness of breath, sore throat, coryza). Contacts/epidemiological links not taken into consideration. ^2^ Individuals with fever OR anosmia/ageusia AND at least one respiratory symptom (dry cough, productive cough, shortness of breath, sore throat, coryza). Contacts/epidemiological links not taken into consideration. * Chi-squared tests.

**Table 6:** Logistic Regression with Generalized Estimation Equations (GEE) estimation procedure for Composite Adherence score dichotomized as adherence (coded as 1) and non-adherence (coded as 0).

Covariates	Adjusted OR (95% CI)	*p*-Value
Age	0.99 (0.98–0.99)	0.026
Gender		
Male	Ref	
Female	1.01 (0.85–1.19)	0.942
Maximum education level		
University/Postgraduate	Ref	
Primary/Secondary	0.60 (0.46–0.78)	<0.001
What do you do for a living?		
Jobless/Student	Ref	
Companies	2.31 (1.66–3.22)	<0.001
Government	1.61 (1.04–2.49)	0.032
Self-employed	0.86 (0.70–1.07)	0.168
What are your housing conditions?		
Homeless	Ref	
Hut	0.44 (0.11–1.73)	0.238
Room	0.36 (0.15–0.89)	0.027
Studio	0.26 (0.11–0.61)	0.002
Apartment	0.29 (0.10–0.82)	0.019
Villa	0.26 (0.05–1.41)	0.119
Healthcare worker		
No	Ref	
Yes	2.19 (1.57–3.05)	<0.001

OR = Odd ratios, GEE = Generalized Estimation Equations, Number of clusters = 7, Maximum cluster size = 689.

## Data Availability

Data are available upon reasonable request. Data are available on the International Consortium (International Citizen Project COVID-19 (ICPcovid): http://www.icpcovid.com, accessed on 03 January 2021) website and could be used by other investigators on request. De-identified participant data are available.

## Supplementary Materials

The following are available online at https://www.mdpi.com/1660-4601/18/5/2525/s1, RDC Covid-19 Questionnaire.

## References

[1] World Health Organization (2021). WHO Coronavirus Disease (COVID-19) Dashboard.

[2] European Centre for Disease Prevention and Control (2021). COVID-19 Situation Update Worldwide, as of 13 January 2021. https://www.ecdc.europa.eu/en/geographical-distribution-2019-ncov-cases.

[3] World Health Organization (2020). Archived: WHO Timeline—COVID-19.

[4] Wang Q., Yu C. (2020). The role of masks and respirator protection against SARS-CoV-2. Infect. Control. Hosp. Epidemiol..

[5] Noh J.Y., Seong H., Yoon J.G., Song J.Y., Cheong H.J., Kim W.J. (2020). Social Distancing against COVID-19: Implication for the Control of Influenza. J. Korean Med. Sci..

[6] Jang W.M., Jang D.H., Lee J.Y. (2020). Social Distancing and Transmission-reducing Practices during the 2019 Coronavirus Disease and 2015 Middle East Respiratory Syndrome Coronavirus Outbreaks in Korea. J. Korean Med. Sci..

[7] Bustamante-Castañeda F., Caputo J.-G., Cruz-Pacheco G., Knippel A., Mouatamide F. (2021). Epidemic model on a network: Analysis and applications to COVID-19. Phys. A Stat. Mech. Appl..

[8] Tayech A., Mejri M.A., Makhlouf I., Mathlouthi A., Behm D.G., Chaouachi A. (2020). Second Wave of COVID-19 Global Pandemic and Athletes’ Confinement: Recommendations to Better Manage and Optimize the Modified Lifestyle. Int. J. Environ. Res. Public Health.

[9] Ghanbari B. (2020). On forecasting the spread of the COVID-19 in Iran: The second wave. Chaos Solitons Fractals.

[10] Polack F.P., Thomas S.J., Kitchin N., Absalon J., Gurtman A., Lockhart S., Perez J.L., Marc G.P., Moreira E.D., Zerbini C. (2020). Safety and Efficacy of the BNT162b2 mRNA Covid-19 Vaccine. N. Engl. J. Med..

[11] Walsh E.E., Frenck R.W., Falsey A.R., Kitchin N., Absalon J., Gurtman A., Lockhart S., Neuzil K., Mulligan M.J., Bailey R. (2020). Safety and Immunogenicity of Two RNA-Based Covid-19 Vaccine Candidates. N. Eng. J. Med..

[12] Ramasamy M.N., Minassian A.M., Ewer K.J., Flaxman A.L., Folegatti P.M., Owens D.R., Voysey M., Aley P.K., Angus B., Babbage G. (2020). Safety and immunogenicity of ChAdOx1 nCoV-19 vaccine administered in a prime-boost regimen in young and old adults (COV002): A single-blind, randomised, controlled, phase 2/3 trial. Lancet.

[13] Ditekemena J., Doumbia S., Ebrahim S.H. (2020). COVID-19′s final frontier: The central Africa region. Travel Med. Infect. Dis..

[14] Ditekemena J. (2020). COVID-19 amidst Ebola’s retreat. Science.

[15] Ditekemena J., Nkamba D., Muhindo M.H., Nelson Siewe Fodjo J.N., Luhata C., Van den Bergh R., Tshefu Kitoto A., Van Damme W., Muyembe J.J., Colebunders R. (2020). Factors associated with adherence to COVID-19 prevention measures in the Democratic Republic of Congo (DRC): Results of an online survey. BMJ Open.

[16] Claude K.M., Underschultz J., Hawkes M.T. (2018). Ebola virus epidemic in war-torn eastern DR Congo. Lancet.

[17] Agresti A. (2007). An Introduction to Categorical Data Analysis.

[18] World Health Organization (2020). Diagnostic Testing for SARS-CoV-2.

[19] World Health Organization (2020). WHO COVID-19 Case Definition, Updated in Public Health Surveillance for COVID-19.

[20] Li T., Liu Y., Li M., Qian X., Dai S.Y. (2020). Mask or no mask for COVID-19: A public health and market study. PLoS ONE.

[21] Konda A., Prakash A., Moss G.A., Schmoldt M., Grant G.D., Guha S. (2020). Aerosol Filtration Efficiency of Common Fabrics Used in Respiratory Cloth Masks. ACS Nano.

[22] Fodjo J.N.S., Pengpid S., Villela E.F.D.M., Van Thang V., Ahmed M., Ditekemena J., Crespo B.V., Wanyenze R.K., Dula J., Watanabe T. (2020). Mass masking as a way to contain COVID-19 and exit lockdown in low- and middle-income countries. J. Infect..

[23] Zogning Makemjio E., Tiotsia Tsapi A., Défo Tamgno E., Djeunang Dongho G.B., Nguefack-Tsague G., Montesano C., Colizzi V., Russo G., Sanou Sobze M. (2020). Knowledge and Attitudes of Population Living in Rural and Semi-Rural Areas towards Covid-19: Case of the Menoua Division, Cameroon. Ig. Sanita Pubbl..

[24] Belingheri M., Paladino M.E., Riva M.A. (2020). COVID-19: Health prevention and control in non-healthcare settings. Occup. Med..

[25] Basu S. (2020). Non-communicable disease management in vulnerable patients during Covid-19. Indian J. Med. Ethic.

[26] Wada K., Higuchi Y., Smith D.R. (2015). Socioeconomic status and self-reported health among middle-aged Japanese men: Results from a nationwide longitudinal study. BMJ Open.

[27] Corburn J., Vlahov D., Mberu B., Riley L., Caiaffa W.T., Rashid S.F., Ko A., Patel S., Jukur S., Martínez-Herrera E. (2020). Slum Health: Arresting COVID-19 and Improving Well-Being in Urban Informal Settlements. J. Urban Health.

[28] Rowe A.K., Rowe S.Y., Peters D.H., Holloway K.A., Chalker J., Ross-Degnan D. (2018). Effectiveness of strategies to improve health-care provider practices in low-income and middle-income countries: A systematic review. Lancet Glob. Health.

[29] Chu D.K., Akl E.A., Duda S., Solo K., Yaacoub S., Schünemann H.J., El-Harakeh A., Bognanni A., Lotfi T., Loeb M. (2020). Physical distancing, face masks, and eye protection to prevent person-to-person transmission of SARS-CoV-2 and COVID-19: A systematic review and meta-analysis. Lancet.

