# Supplementary material for: Adherence to COVID-19 Prevention Measures in the Democratic Republic of the Congo, Results of Two Consecutive Online Surveys
Source: Int J Environ Res Public Health. 2021 Mar 4;18(5):2525. doi: 10.3390/ijerph18052525 (PMC7967309; doi:10.3390/ijerph18052525)
Supplement: Supplementary file 1 [file ijerph-18-02525-s001.pdf]

RDC Covid-19 Questionnaire

Socio-demographic information

Q: Age (*)

Type: number

A: number (min: 1 / max: 110 / step: 1)

Q: Sex (*)

Type: choice

A: one of the following:

male	=>	*Male*
female	=>	*Female*

Q: Nationality (*)

Type: choice

A: one of the following:

local	=>	*Congolese*
foreigner	=>	*Foreigner*

Q: In which province of Congo do you live? (*)

Type: choice

A: one of the following:

bas_uele	=>	*Bas-Uele*
equateur	=>	*Équateur*
haut_katanga	=>	*Haut-Katanga*
haut_lomami	=>	*Haut-Lomami*
haut-uele	=>	*Haut-Uele*
ituri	=>	*Ituri*
kasaï	=>	*Kasaï*
kasaï_central	=>	*Kasaï-Central*
kasaï_oriental	=>	*Kasaï-Oriental*
kinshasa	=>	*Kinshasa*
kongo_central	=>	*Kongo-Central*
kwango	=>	*Kwango*
kwilu	=>	*Kwilu*
lomami	=>	*Lomami*
lualaba	=>	*Lualaba*
mai_ndombe	=>	*Mai-Ndombe*
maniema	=>	*Maniema*
mongala	=>	*Mongala*
nord_kivu	=>	*Nord-Kivu*

nord_ubangi	=>	*Nord-Ubangi*
sankuru	=>	*Sankuru*
sud_kivu	=>	*Sud-Kivu*
sud_ubangi	=>	*Sud-Ubangi*
tanganyika	=>	*Tanganyika*
tshopo	=>	*Tshopo*
tshuapa	=>	*Tshuapa*

Q: Religion (*)

Type: choice

A: one of the following:

protestant	=>	*Protestant*
catholic	=>	*Catholic*
pentecostal	=>	*Pentecostal*
adventist	=>	*Seventh Day Adventist*
muslim	=>	*Muslim*
jehovah_witness	=>	*Jehovah Witness*
other	=>	*Other*
none	=>	*None*

Q: Highest educational level (*)

Type: choice

A: one of the following:

primary	=>	*Primary*
Secondary	=>	*Secondary*
University Undergraduate degree holder	=>	*University Undergraduate degree holder*
University Postgraduate degree holder	=>	*University Postgraduate degree holder*

Q: Marital status (*)

Type: choice

A: one of the following:

single	=>	*Single*
legally_married	=>	*Legally married*
cohabitation	=>	*Cohabitation*
divorced	=>	*Divorced*
widow_widower	=>	*Widow/widower*

Q: Who do you currently live with? (many answers possible) (*)

Type: choice_multiple

A: multiple answers possible:

My parent(s)	=>	*My parent(s)*
My spouse/partner	=>	*My spouse/partner*
My child(ren)	=>	*My child(ren)*
My sibling(s) or other relative(s)	=>	*My sibling(s) or other relative(s)*
Friends	=>	*Friends*
I live alone	=>	*I live alone*

How many housemates do you have (yourself not included)? Please write the number of persons corresponding to each age group. Write 0 if there is nobody in a given age group.

Q: Adults over 70 years of age: (*)

Type: number

A: number (min: n/a / max: 10 / step: n/a)

Q: Adults between 18 and 70 years: (*)

Type: number

A: number (min: n/a / max: 10 / step: n/a)

Q: Children between 12 and 17 years: (*)

Type: number

A: number (min: n/a / max: 10 / step: n/a)

Q: Children younger than 12 years: (*)

Type: number

A: number (min: n/a / max: 10 / step: n/a)

Q: Do you live in: (*)

Type: choice

A: one of the following:

rural_area	=>	*a rural area/village?*
suburb	=>	*a suburban area?*
urban_town	=>	*an urban area/town*
other	=>	*Other (specify)*

Q: Specify urban area: (*)

Type: choice

A: one of the following:

headquarter_region	=>	*In a regional headquarter*
capital_country	=>	*In the capital of the country*
economic_capital	=>	*In the economic capital*
big_city	=>	*In another big city*

Visible if

Q:	A:
Do you live in:	- value => urban_town

Q: Specify where you live: (*)

Type: text

A: text input

Visible if

Q:	A:
Do you live in:	- value => other

Q: What are your housing conditions? (*)

Type: choice

A: one of the following:

room	=>	*a room*
studio	=>	*a studio (a room, with kitchen and toilet)*
apartment	=>	*an apartment with many other households in the same building*
villa	=>	*a villa*
hut	=>	*a hut*
homeless	=>	*homeless*

Q: In the context of your country, what category is most appropriate for your current socio-economic situation?(*)

Type: choice

A: one of the following:

low_income	=>	*Low income category*
low_middle	=>	*Lower middle income category*
upper_middle	=>	*Upper middle income category*
high_income	=>	*High income category*

Daily life during the coronavirus epidemic

Q: How do you obtain information about the coronavirus epidemic? (many answers possible) (*)

Type: choice_multiple

A: multiple answers possible:

family_neighbours	=>	*From family, neighbours or friends*
radio	=>	*From the radio*
television	=>	*From the television*
social_media	=>	*From the social media (WhatsApp, Facebook, Twitter, etc)*
government	=>	*From government authorities*
chief	=>	*From the village/quarter chief*
religious	=>	*From the religious authorities*
health_personnel	=>	*From healthcare workers (nurses, doctors, etc)*

Q: How many people apart from your housemates did you talk to yesterday face to face (not by phone, chat etc)?(*)

Type: number

A: number (min: n/a / max: n/a / step: n/a)

Q: When was the last time you shook hands, hugged, gave a kiss or had any form of physical contact with someone other than a housemate, during the last month? (*)

Type: choice

A: one of the following:

today	=>	*Today*
last_two_days	=>	*Last two days*
last_3_to_6_days	=>	*Last 3 to 6 days*
one_to_two_weeks	=>	*Between one to two weeks ago*
more_than_two_weeks	=>	*More than two weeks ago*
no_contacts	=>	*No contacts with persons outside my household during the last month*

Q: During the last week did you have difficulties in obtaining food or essential needs??(*)

Type: choice

A: one of the following:

Yes	=>	*Yes*
No	=>	*No*

Q: What was the most important reason you had difficulties in obtaining food last week?(*)

Type: choice

A: one of the following:

no_money	=>	*No money due to lost revenue since the coronavirus epidemic started*
little_food_available	=>	*Little food available in shops/market*
expensive	=>	*Food has become too expensive since the coronavirus epidemic started*
unsafe_to_go_out	=>	*I felt it was unsafe to go out to buy food*
to_ill_to_go_out	=>	*I was too ill to go out*

Visible if

Q:	A:
During the last week did you have difficulties in obtaining food or essential needs??	- value => Yes

Q: During the last week, how worried or afraid were you about your health?(*)

Type: choice_scale

A: 1 = not worried to 5 = extremely worried

1	=>	*1*
2	=>	*2*
3	=>	*3*
4	=>	*4*
5	=>	*5*

Q: Have you suffered any form of violence or discrimination because of the measures taken against the coronavirus? (many answers possible) (*)

Type: choice_multiple

A: multiple answers possible:

home_violence	=>	*Physical violence at home*
outside_violence	=>	*Physical violence outside*
socio_economic_discrimination	=>	*Discrimination because of my social/economic status*
quarantined	=>	*Discrimination because I was quarantined*
ethnic_discrimination	=>	*Discrimination because of my ethnicity*
no_violence_discrimination	=>	*No violence or discrimination*

Q: During the COVID-19 lockdown, have you experienced violence from your sexual partner (if applicable)?(*)

Type: choice

A: one of the following:

yes	=>	*Yes*
no	=>	*No*
not_applicable	=>	*Not applicable*

Q: If yes, which kind of violence did you experience? (*)

Type: choice

A: one of the following:

verbal	=>	*Verbal violence*
physical	=>	*Physical violence*
sexual	=>	*Sexual violence, including rape*
other	=>	*Other*

Visible if

Q:	A:
During the COVID-19 lockdown, have you experienced violence from your sexual partner (if applicable)?	- value => yes

Q: How did you arrange the care of the kids in your home today?(*)

Type: choice

A: one of the following:

home_by_myself	=>	*At home, by myself*
home_housemates	=>	*At home, with my housemates*
school_childcare	=>	*To school / childcare*
friends_acquaintances	=>	*At friends / acquaintances*
aunts_uncles	=>	*At aunts or uncles*
grandparents	=>	*At grandparents*
home_house_help	=>	*At home, with a house help/nanny*
other	=>	*Other*
not_applicable	=>	*Not Applicable (no children at home)*

Professional life during the coronavirus epidemic

Q: What do you do for a living? (*)

Type: choice

A: one of the following:

student	=>	*Student*
jobless	=>	*Jobless*
self_employed	=>	*Self-employed*
company	=>	*Work for a person, institution or company*
government	=>	*Work for the government*

Q: Are you a healthcare worker or a student working in the health sector? (*)

Type: choice

A: one of the following:

yes_worker	=>	*Yes, I am a healthcare worker*
yes_student	=>	*Yes, I am a healthcare student*
no	=>	*No*

Q: If yes, what is your main function in the health structure? (*)

Type: choice

A: one of the following:

nurse	=>	*Nurse*

gp	=>	*Medical doctor: general practitioner*
specialist	=>	*Medical doctor: resident/specialist*
lab	=>	*Laboratory staff*
pharmacy	=>	*Pharmacy staff*
research_maintenance	=>	*Research/Maintenance*
admin_finance	=>	*Administration/finance*
technician	=>	*Technician (imaging, specialized services, etc)*

Visible if

Q:	A:
0	- :input[name="are_you_a_healthcare_worker_or_a_student_working_in_the_health_s"] =>
1	
2	- :input[name="are_you_a_healthcare_worker_or_a_student_working_in_the_health_s"] =>

Q: Have you been given any protective equipment at your place of work? (many answers possible)(*)

Type: choice_multiple

A: multiple answers possible:

masks	=>	*Face masks*
gloves	=>	*Gloves*
shield	=>	*Face shield*
boots	=>	*Boots*
apron	=>	*Full protective apron*
gel	=>	*Hand gels/sanitizers*
none	=>	*None*

Visible if

Q:	A:
0	- :input[name="are_you_a_healthcare_worker_or_a_student_working_in_the_health_s"] =>
1	
2	- :input[name="are_you_a_healthcare_worker_or_a_student_working_in_the_health_s"] =>

Q: What are your current working conditions? (*)

Type: choice

A: one of the following:

worker_from_home	=>	*Worker from home*
worker_open_space	=>	*Worker in an open space (market, shop, roadside, etc)*
worker_closed_space_alone	=>	*Worker in a closed indoor space alone (office, …., etc.)*
worker_closed_space_people	=>	*Worker in a closed indoor space with several people (office, …., etc.)*
not_applicable	=>	*Not applicable (if jobless or student)*

Q: How many days per week do you usually go to work? (*)

Type: number

A: number (min: n/a / max: 7 / step: n/a)

Q: How many days did you (physically) go to work last week? (*)

Type: number

A: number (min: n/a / max: 7 / step: n/a)

Q: Are you exclusively working from home this week? (*)

Type: choice

A: one of the following:

yes	=>	*Yes*
no	=>	*No*
not_applicable	=>	*Not applicable (if jobless or student)*

Q: Why are you not working from home? (*)

Type: choice

A: one of the following:

not_possible	=>	*It is not possible with my job*
not_allowed	=>	*It is possible, but is not allowed by my employer*
home_not_working	=>	*I am at home but not working*
no_risk_to_go_out	=>	*I don’t think there is any risk to go out*
other	=>	*Other*

Visible if

Q:	A:
Are you exclusively working from home this week?	- value => no

Q: What transportation means did you use to go to work? (*)

Type: choice

A: one of the following:

public_transport	=>	*By public transport with multiple people (taxi, bus, motorcycle, etc)*
hired_vehicle	=>	*Hired a vehicle for myself and/or family members (private taxi, rented car, etc)*
own_transport	=>	*personal vehicle (Car, motorcycle, etc)*
walked	=>	*Walked to work*

Visible if

Q:	A:
Are you exclusively working from home this week?	- value => no

Personal preventive measures for coronavirus

Q: I follow the social 1.5-2m meters distance rule (*)

Type: choice

A: one of the following:

Yes	=>	*Yes*
No	=>	*No*

Q: I wear a face mask when going outside (*)

Type: choice

A: one of the following:

yes	=>	*Yes*
no	=>	*No*
na	=>	*Not applicable (I do not go out)*

Q: If yes, when/where do you wear a face mask? (many answers possible) (*)

Type: choice_multiple

A: multiple answers possible:

home	=>	*At home*
work	=>	*At work*
sometimes_out	=>	*Sometimes when I go out*
always_out	=>	*Everytime I go out*

Visible if

Q:	A:
I wear a face mask when going outside	- value => yes

Q: If no, what is (are) the reason(s) for not using face masks? (many answers possible) (*)

Type: choice_multiple

A: multiple answers possible:

no_mask	=>	*I do not have a mask*
no_money	=>	*I don't have money to buy masks*
where_to_get	=>	*I don't know where to get a mask*
not_needed	=>	*I don't think that masks are needed*

Visible if

Q:	A:
I wear a face mask when going outside	- value => no

Q: When I cough or sneeze, I do so in my elbow and/or I cover my mouth with a tissue paper(*)

Type: choice

A: one of the following:

Yes	=>	*Yes*
No	=>	*No*

Q: When I cough or sneeze, I usually wash/desinfect my hands immediately afterwards (*)

Type: choice

A: one of the following:

Yes	=>	*Yes*
No	=>	*No*

Q: I measure my body temperature at least twice a week (*)

Type: choice

A: one of the following:

Yes	=>	*Yes*
No	=>	*No*

Q: I wash my hands using soap and water regularly during the day (*)

Type: choice

A: one of the following:

Yes	=>	*Yes*
No	=>	*No*

Q: I use a hand sanitizer regularly during the day (*)

Type: choice

A: one of the following:

Yes	=>	*Yes*
No	=>	*No*

Q: What constraints to hand washing/use of hand sanitizers do you face?

Type: text

A: text input

Visible if

Q:	A:
I wash my hands using soap and water regularly during the day	- value => No
I use a hand sanitizer regularly during the day	- value => No

Q: I avoid touching my face (eyes, nose and mouth) (*)

Type: choice

A: one of the following:

Yes	=>	*Yes*
No	=>	*No*

Q: I disinfect my phone whenever I return home (*)

Type: choice

A: one of the following:

yes	=>	*Yes*
no	=>	*No*
na	=>	*Not applicable*

Q: I stay home when I feel flu-like symptoms (*)

Type: choice

A: one of the following:

Yes	=>	*Yes*
No	=>	*No*

Q: On a scale of 1 to 5, how difficult is it for you personally to follow the protective measure of staying home as much as possible? (*)

Type: choice_scale

A: 1 = not difficult at all; 5 = extremely difficult

1	=>	*1*
2	=>	*2*
3	=>	*3*
4	=>	*4*
5	=>	*5*

Community preventive measures for coronavirus

Q: Were you in a meeting or gathering with with 50 persons or more during the last 7 days?(*)

Type: choice

A: one of the following:

Yes	=>	*Yes*
No	=>	*No*

Q: What was the nature of the gathering? (multiple answers possible) (*)

Type: choice_multiple

A: multiple answers possible:

restaurant	=>	*Restaurant*
bar	=>	*Bar*
club	=>	*Club*
party	=>	*Party*
funeral	=>	*Funeral*
religious	=>	*Religious gathering*
family	=>	*Family gathering*
sport	=>	*Sports event*
other	=>	*Other*

Visible if

Q:	A:
Were you in a meeting or gathering with with 50 persons or more during the last 7 days?	- value => Yes

Q: Were you in a car with 4 or more other persons during the last 7 days?(*)

Type: choice

A: one of the following:

Yes	=>	*Yes*
No	=>	*No*

Q: Were you in a public gym in the past 7 days ? (*)

Type: choice

A: one of the following:

Yes	=>	*Yes*
No	=>	*No*

Q: Did you go to a barber/hairdresser, beauty parlour, massages, spa, or nail studio in the past 7 days ?(*)

Type: choice

A: one of the following:

Yes	=>	*Yes*
No	=>	*No*

Q: Did you go to a market in the past 7 days ? (*)

Type: choice

A: one of the following:

Yes	=>	*Yes*
No	=>	*No*

Q: Did you travel in the past 7 days? (*)

Type: choice

A: one of the following:

yes_other_regions	=>	*Yes, I travelled to other regions of the country*
yes_outside_country	=>	*Yes, I travelled outside of the country*
no_travel	=>	*No travel*

Q: During the last week, how worried or afraid were you about the health of your loved ones?(*)

Type: choice_scale

A: 1 = not worried to 5 = extremely worried

1	=>	*1*
2	=>	*2*
3	=>	*3*
4	=>	*4*
5	=>	*5*

Q: On a scale of 1 to 10, can you indicate the extent to which people in your environment have practically adapted their behavior to the government recommendations? (*)

Type: choice_scale

A: 1 = no adaptions, 10 = very strong adaptation

1	=>	*1*
2	=>	*2*
3	=>	*3*
4	=>	*4*
5	=>	*5*
6	=>	*6*
7	=>	*7*
8	=>	*8*
9	=>	*9*
10	=>	*10*

Questions related to your personal health

Q: Have you been eating more healthy food such as fruits and vegetables since the coronavirus epidemic started? (*)

Type: choice

A: one of the following:

Yes	=>	*Yes*

No	=>	*No*

Q: Have you been taking more vitamin tablets since the coronavirus epidemic started? (*)

Type: choice

A: one of the following:

Yes	=>	*Yes*
No	=>	*No*

Q: Did you experience any of the following flu-like symptoms during the last 14 days? (multiple options possible)(*)

Type: choice_multiple

A: multiple answers possible:

fever	=>	*Fever*
headaches	=>	*Headaches*
sore_throat	=>	*Sore throat*
loss_taste	=>	*Loss of taste*
loss_smell	=>	*Loss of smell*
stuffy_nose	=>	*Stuffy and/or running nose*
dry_cough	=>	*Dry cough*
productive_cough	=>	*Productive cough*
shortness_breath	=>	*Shortness of breath*
muscle_pain	=>	*Muscle or body pains*
weakness	=>	*General weakness*
nausea	=>	*Nausea*
diarrhea	=>	*Diarrhea*
none	=>	*None of the above*

Q: For how many days did you have flu-like symptoms? (*)

Type: number

A: number (min: 1 / max: 50 / step: n/a)

Visible if

Q:	A:
:input[name="if_yes_which_symptoms_did_you_experience_multiple_options_possib[none]"]	- unchecked => 1

Q: Have any of your housemates had flu-like symptoms in the last 14 days? (*)

Type: choice

A: one of the following:

yes	=>	*Yes*
no	=>	*No*

do_not_know	=>	*Do not know*

Q: Have you been tested for the coronavirus? (*)

Type: choice

A: one of the following:

yes	=>	*Yes*
no	=>	*No*

Q: If yes, what was the result of the test? (*)

Type: choice

A: one of the following:

positive	=>	*Positive*
negative	=>	*Negative*
do_not_know	=>	*Do not know yet*

Visible if

Q:	A:
Have you been tested for the coronavirus?	- value => yes

Q: When was your coronavirus test done? (*)

Type: choice

A: one of the following:

two_weeks	=>	*During the past two weeks*
one_month	=>	*Between two weeks and one month*
more_than_one_month	=>	*More than one month ago*

Visible if

Q:	A:
Have you been tested for the coronavirus?	- value => yes

Q: Do you smoke? (*)

Type: choice

A: one of the following:

Yes	=>	*Yes*
No	=>	*No*

Q: Do you have any of the following chronic/underlying disease? (many answers possible) (*)

Type: choice_multiple

A: multiple answers possible:

heart_disease	=>	*Heart disease*
hypertension	=>	*Hypertension*
diabetes	=>	*Diabetes*
cancer	=>	*Cancer*
hiv	=>	*HIV*
tb	=>	*Tuberculosis*
asthma	=>	*Asthma*
other	=>	*Other*
none	=>	*None of the above*

Q: If you have an underlying disease did you experience difficulties to obtain your medication since the COVID outbreak started? (*)

Type: choice

A: one of the following:

Yes	=>	*Yes*
No	=>	*No*

Visible if

Q:	A:
:input[name="do_you_have_any_of_the_following_underlying_disease_many_answers[none]"]	- unchecked => 1

Q: In your opinion, is the coronavirus lockdown necessary in Congo? (*)

Type: choice

A: one of the following:

yes	=>	*Yes*
no	=>	*No*

Q: I fully understand what this study is about, and I freely consent to participate. All the information I provide can be used by researchers to better understand coronavirus disease in Congo. (*)

Type: checkbox

A: checkbox
